# Protective and therapeutic effects of hexagonal boron nitride against hydrogen peroxide-induced oxidative damage in human gingival fibroblasts

**DOI:** 10.1186/s12903-025-07010-1

**Published:** 2025-10-14

**Authors:** Isil Yalcin Bilke, Bilge Meraci Yildiran, Akif Hakan Kurt, Cansu Kara Oztabag, Nese Aysit, Yapıncak Goncu, Mehmet Ali Sungur

**Affiliations:** 1 Department of Periodontology, Faculty of Dentistry, Bolu Abant Izzet Baysal University, Bolu, 14030 Türkiye; 2 Department of Medicinal Pharmacology, Faculty of Medicine, Bolu Abant Izzet Baysal University, Bolu, 14280 Türkiye; 3 Department of Medical Biology, School of Medicine, Health Science and Technologies Research Institute (SABITA), Istanbul Medipol University, Istanbul, 34815 Türkiye; 4 Department of Biomedical Engineering, Engineering Architecture Faculty, Eskisehir Osmangazi University, Eskisehir, 26480 Türkiye; 5 Biostatistics and Medical Informatics, Faculty of Medicine, Duzce University, Duzce, 81620 Türkiye

**Keywords:** Cell viability, Fibroblasts, Hexagonal boron nitride, Oxidative stress

## Abstract

**Background:**

Oxidative stress plays a significant role in the progression of periodontal diseases. Hexagonal boron nitride (hBN) nanoparticles have antimicrobial, antiplaque, and antioxidant properties. The aim of this study to evaluate the protective and therapeutic effects of hBN against oxidative stress induced by hydrogen peroxide (H_2_O_2_) in human gingival fibroblast (HGF) cells.

**Methods:**

In this study, a primary cell line of HGFs was used. Oxidative stress was induced by treating the cells with 800 µM H_2_O_2_. hBN was applied at concentrations of 0.001, 0.005, and 0.01 mg/mL, either 2 h prior to or 2 h following H₂O₂ exposure. Cells were incubated for 24 h post-treatment. Cell viability was assessed using the 2,3-bis-[2-methoxy-4-nitro-5-sulfophenyl]-2 H-tetrazolium-5-carboxyanilide salt (XTT) assay. Additionally, the total oxidant status (TOS) was measured before and after oxidative damage at the 0.01 mg/mL hBN concentration.

**Results:**

hBN did not negatively affect cell viability in HGF cells at any concentration. The application of hBN to HGF cells before and after oxidative damage resulted in statistically significant (*P* < 0.001), albeit modest improvements in cell viability (2-5.5% and 3-6.5%, respectively). No significant differences were observed in TOS (*P* = 0.234), indicating that the cytoprotective effects were not mediated via alterations in TOS.

**Conclusions:**

Within the limitations of this in vitro study, hBN was found to enhance cell viability under H₂O₂-induced oxidative stress in HGF cells. These findings suggest a potential role for hBN in modulating antioxidant responses and supporting periodontal healing processes. However, their clinical relevance remains uncertain. Therefore, further mechanistic studies and clinical investigations are required to validate the potential adjunctive role of hBN in the prevention and treatment of periodontal diseases.

## Introduction

Periodontal disease, widely recognized as one of the most prevalent oral health conditions, is characterized by inflammation that occurs when the supportive tissues surrounding the teeth are compromised. A significant aspect of this condition involves the interplay between host responses and bacterial activity [1, 2]. Recent studies have increasingly highlighted the role of oxidative stress in the pathogenesis of chronic inflammatory diseases, including periodontitis [3]. Oxidative stress refers to a state where there is an imbalance between the production of reactive oxygen species (ROS) and the body’s endogenous antioxidant defenses. As periodontitis advances, neutrophils become hyperactivated, resulting in the secretion of ROS, such as hydrogen peroxide (H_2_O_2_) and superoxide, in response to bacterial presence. The elevated levels of ROS contribute to increased lipid peroxidation, along with damage to DNA and proteins, ultimately leading to cellular and tissue injury [4, 5]. Moreover, ROS act as pivotal signaling molecules that activate the nuclear factor (NF)-κB signaling pathway, facilitating the expression of pro-inflammatory factors. In addition, ROS are implicated in the differentiation and activation of osteoclasts through the receptor activator of NF-κB ligand (RANKL) signaling pathway, which results in alveolar bone resorption and the degradation of periodontal tissue [6, 7].

Initial periodontal treatment, which serves as the cornerstone for managing periodontitis, typically encompasses scaling and root planing. While this approach yields clinical success in the majority of periodontitis cases, certain complex clinical scenarios may not show complete improvement [8, 9]. Various adjunctive therapies and pharmacological agents have been used to improve the outcomes of periodontal treatment, with chlorhexidine being one of the most commonly used antimicrobial agents. However, in addition to their benefits, these agents also present several disadvantages [10–15]. For example, chlorhexidine is known for its broad-spectrum antimicrobial activity and strong substantivity, yet its long-term use may lead to undesirable effects such as tooth and tongue staining, altered taste sensation, mucosal irritation, and cytotoxicity at higher concentrations [10, 15]. Systemic antibiotics such as amoxicillin and metronidazole are effective in specific cases but may cause gastrointestinal disturbances, hypersensitivity reactions, and contribute to the development of antimicrobial resistance [16, 17]. Locally delivered agents like minocycline microspheres and doxycycline gels offer targeted delivery with reduced systemic exposure; however, their high cost and limited clinical efficacy may restrict routine use [18, 19]. Given these drawbacks, there is an ongoing demand for safer, more biocompatible, and multifunctional alternatives in periodontal therapy.

Boric acid (BA), a boron-derived compound has gained attention as a potential adjunct in periodontal therapy [20–22]. Research conducted on pre-clinical as well as clinical studies has demonstrated that BA can positively influence treatment outcomes by mitigating periodontal inflammation and reducing attachment loss. The therapeutic effects of boron-containing compounds are attributed to their antimicrobial, anti-inflammatory, and immunomodulatory properties [20, 21, 23]. Clinical investigations have indicated that procedures such as subgingival irrigation with 0.75% BA or the application of a 0.75% BA gel, when used in conjunction with mechanical periodontal treatment, yield improvements in periodontal clinical parameters among patients with periodontitis. These findings suggest that BA may serve as a viable alternative to chlorhexidine in periodontal therapy [22, 24]. Another notable boron-containing compound is boron nitride (BN), a laboratory-synthesized material composed of equal numbers of B and N atoms [25–27]. While it is not found in nature in its free form, BN can manifest in various crystalline structures, which are influenced by alterations in pressure and temperature. Hexagonal boron nitride (hBN) is the most stable form at room temperature [28, 29]. Among boron-based compounds, hBN nanoparticles stands out due to its remarkable physicochemical properties, including low density, high thermal conductivity, chemical stability, and mechanical strength. Importantly, hBN also demonstrates favorable biological characteristics, such as high biocompatibility and low cytotoxicity, which distinguish it from other boron-containing substances [30–35]. Due to this unique combination of chemical and biological features, hBN has attracted growing interest for use in biomedical applications [31–33].

In a study investigating the antimicrobial and antibiofilm properties of hBN nanoparticles, it was found that hBN exhibited pronounced antibiofilm activity against preformed biofilms and effectively inhibited the growth of certain strains of *Streptococcus mutans* and *Candida* [31]. These findings suggest that hBN holds promise as a potential agent for managing oral biofilms. Furthermore, the study evaluated the cytotoxic effects of hBN nanoparticles on human dermal fibroblasts and Madin-Darby Canine Kidney (MDCK) cells. The results indicated that these nanoparticles exhibited no cytotoxicity at the tested concentrations. Consequently, the researchers proposed that hBN nanoparticles could serve as a safe option for oral care applications, provided they are used at an appropriate concentration, specifically 0.1 mg/mL [31].

Sen et al. conducted a comparative study examining the effects of hBN and its possible degradation product, BA, on wound healing [32]. The findings revealed that both hBN and BA significantly accelerated the wound healing. However, hBN nanoparticles demonstrated superior properties, including slower biodegradation and an extended half-life. The study demonstrated that hBN facilitates wound healing by reducing ROS levels, an effect attributed to its inherent antioxidant properties. Additionally, hBN showed angiogenic activity, stimulated cell proliferation, and protected cells from apoptosis [32]. Cakir et al. investigated the prophylactic effects of hBN nanoparticles on sepsis-induced neurodegeneration [33]. Their findings illustrated that the administration of hBN nanoparticles ameliorated the degenerative effects induced by lipopolysaccharide in a rat model of sepsis. The study reported a reduction in the levels of pro-inflammatory cytokines, specifically tumor necrosis factor (TNF)-α and interleukin (IL)−1β, alongside a decrease in total oxidant status. Concurrently, hBN nanoparticles were shown to enhance total antioxidant status, mitigate oxidative stress, and inhibit apoptosis by decreasing the levels of cytochrome c and caspase-3 [33].

Considering its notable antimicrobial, antiplaque, and antioxidant properties, hBN emerges as a promising biomaterial for the preservation of periodontal health and the management of periodontal diseases [26, 31–33]. Previous studies have demonstrated that BA, a potential degradation product of hBN, exhibits beneficial effects in the treatment of periodontal diseases [20, 22, 24]. However, evidence regarding the biocompatibility of hBN with periodontal cell types, as well as its potential cytoprotective or reparative effects, remains scarce and largely unexplored. Therefore, the objective of the present in vitro investigation was to elucidate the protective and therapeutic effects of hBN application on oxidative stress induced by H_2_O_2_ in human gingival fibroblast (HGF) cells.

## Materials and methods

### Characterization of hBN

The microstructural evaluation of the particles was carried out using a scanning electron microscope (SEM, SUPRA 50VP). SEM imaging provided information on the particle morphology and size distribution. The crystalline structure of the precursor material was analyzed via X-ray diffraction (XRD, Rigaku Miniflex) using CuKα radiation (λ = 1.5406 Å) operated at 40 kV and 15 mA. The diffraction patterns were recorded in the 2θ range of 10°–70° with a scanning rate of 0.5 s⁻¹. The chemical structure and functional groups of the hBN were analyzed using Attenuated Total Reflectance-Fourier Transform Infrared Spectroscopy (ATR-FTIR, Bruker Tensor 27) within the wavenumber range of 4000–700 cm⁻¹. The spectra were recorded with a resolution of 4 cm⁻¹ and averaged over 32 scans.

### Cell culture

Primary human fibroblast cells were obtained from gingival tissue of a single systemically healthy individual who underwent crown lengthening surgery at the Department of Periodontology, Faculty of Dentistry, Bolu Abant Izzet Baysal University. This study was approved by the Clinical Research Ethics Committee of the Bolu Abant Izzet Baysal University (decision number: 2023/208). Before participation, the patient was verbally informed about the study and written informed consent was obtained. The study protocol was conducted in accordance with the Helsinki Declaration.

The excised gingival tissues were transferred into petri dishes inside a laminar flow cabinet and cut into small pieces. Then, they were transferred to 25 cm² flasks and incubated with 2 ml of Dulbecco’s Modified Eagle Medium/Ham’s (DMEM) (Gibco/BRL, Gaithersburg, MD, USA) containing 10% Fetal Bovine Serum (FBS) (Gibco/BRL, Gaithersburg, MD, USA) to cover the tissues. The cell adhesion was monitored using an inverted microscope. When the cells covered approximately one-quarter of the petri dish, the HGF culture was obtained.

HGF cells were cultured in DMEM supplemented with 1% Penicillin-Streptomycin (Gibco/BRL, Gaithersburg, MD, USA), 1% L-glutamine (Hyclone), and 10% FBS at 37 °C with 5% CO_2_. Cell growth was monitored daily using an inverted microscope (Zeiss Microscopy). When the cells reached approximately 75–80% confluence, they were passaged by 1:3 dilution ratio, and all experiments were conducted at the 4th passage.

The oxidative stress-induced inflammation model was created with H_2_O_2_. HGF cells were seeded into 96-well plates at a density of 22.000 cells per well in 200 µL of complete medium and incubated for 24 h. Subsequently, the culture medium was replaced with phenol red-free DMEM containing 0.5% FBS, followed by an additional incubation period of 24 h. A stock H_2_O_2_ solution was prepared by mixing 102 µL of H_2_O_2_ with 10 mL of DMEM. This stock solution was then serially diluted to obtain final concentrations of 100, 200, 400, 600, 800, and 1000 µM [36, 37]. HGF cells were exposed to each concentration for 48 h. Cell viability was assessed using the XTT assay. Based on the results, the concentration of 800 µM was chosen for subsequent experiments, as it consistently caused approximately 35–40% cell death.

For the application of hBN before and after the formation of oxidative damage, HGF cells were seeded into 96-well plates (*n* = 8) at a density of 1.1 × 10⁴ cells per well in equal numbers. The cells were incubated in a humidified incubator (Thermo Scientific) containing 5% CO_2_ and 95% air at 37 °C for 48 h. Solutions of hBN (99.97% purity, BORTEK, Boron Technologies, and Mechatronics Inc., Türkiye) were prepared at various concentrations (0.001, 0.005, 0.01, 0.05, and 0.1 mg/mL), as described in the article by Kivanc et al. [31]. Stock suspensions of hBN were freshly prepared at a concentration of 1 mg/mL in serum-free culture medium (DMEM without FBS) and sonicated for 15 min at room temperature to prevent aggregation/agglomeration before application to the cells. For experimental procedures, hBN was applied at three concentrations (0.001, 0.005, and 0.01 mg/mL), either 2 h before or 2 h after the administration of 100 µL of 800 µM H_2_O_2_ (Merck). The cells were then incubated for 24 h. Following the incubation period, a solution of the 2,3-bis-[2-methoxy-4-nitro-5-sulfophenyl]−2 H-tetrazolium-5-carboxyanilide salt (XTT) (Cell Proliferation Kit II, XTT, Sartorius) was prepared to assess cell viability.

### Cell viability

Cell viability was evaluated with the XTT assay [38]. After adding XTT solution and waiting for 4 h, absorbance values were determined using a microplate reader: $\mathrm{Viability}\left(\%\right)=\left({\mathrm{Absorbance}}_{\mathrm{sample}}/{\mathrm{Absorbance}}_{\mathrm{control}}\right)\times100$ .

At higher concentrations (≥ 0.05 mg/mL), hBN tends to form a dark-colored precipitate that may interfere with spectrophotometric readings in colorimetric assays. This was taken into account during the evaluation of cell viability. To ensure solution stability and avoid interference with spectrophotometric measurements, only concentrations of 0.001, 0.005, and 0.01 mg/mL were used in the subsequent experiments. Experiments were performed with eight biological replicates (*n* = 8), and each well was treated independently and considered a separate biological replicate for statistical analysis.

### Biochemical analysis

Total oxidant status (TOS) were measured in HGF cells using the test kit (Human Total Oxidant Status ELISA Kit Cat.No NN24165O) from Rel Assay Diagnostics according to the manufacturer’s instructions [39]. Biochemical analyses were conducted with six biological replicates (*n* = 6), each treated independently and considered a separate biological replicate for statistical analysis.

### Immunofluorescence analysis

Cells differentiated from HGF cells were seeded onto glass-bottomed Petri dishes. At 48 h post-incubation, the medium was removed, and the cells were washed three times with the phosphate buffered saline (PBS). The cells were then fixed with 4% paraformaldehyde for 15 min. After two additional PBS washes, the cells were incubated for 30 min at room temperature in blocking solution (3% BSA, 0.1% Sodium Azide, 0.1% Tween 20). Cells were incubated with 488-conjugated phalloidin (Thermo Fisher, A12379) for 2 h at 4 °C. The 4′,6-diamidino-2-phenylindole (DAPI) was added, and the cells were incubated for an additional 5 min. After washing with PBS containing 0.1% Tween 20, images were captured using an LSM 800 confocal microscope with ZEN BLUE software at 20x magnification.

### Statistical analysis

Statistical analyses were performed with SPSS software package, version V22.0 for Windows (SPSS Inc., Chicago, IL, USA). The Shapiro-Wilk test was employed to determine whether the data exhibited a normal distribution, and the kurtosis and skewness coefficients were analyzed as well. Homogeneity of variances across groups was examined using Levene’s test. One-way ANOVA was carried out to identify differences among the groups, with Tukey’s HSD post hoc test applied for multiple comparisons. Furthermore, the Dunnett’s post-hoc test was used for comparing each application only with the control group. Two-way ANOVA was employed to examine potential hBN application time × group interactions, and Bonferroni adjuested post hoc tests were used for within-group changes and multiple comparisons when needed. A *P* value < 0.05 was considered significant.

## Results

### hBN characterization results

**Fig. 1:**
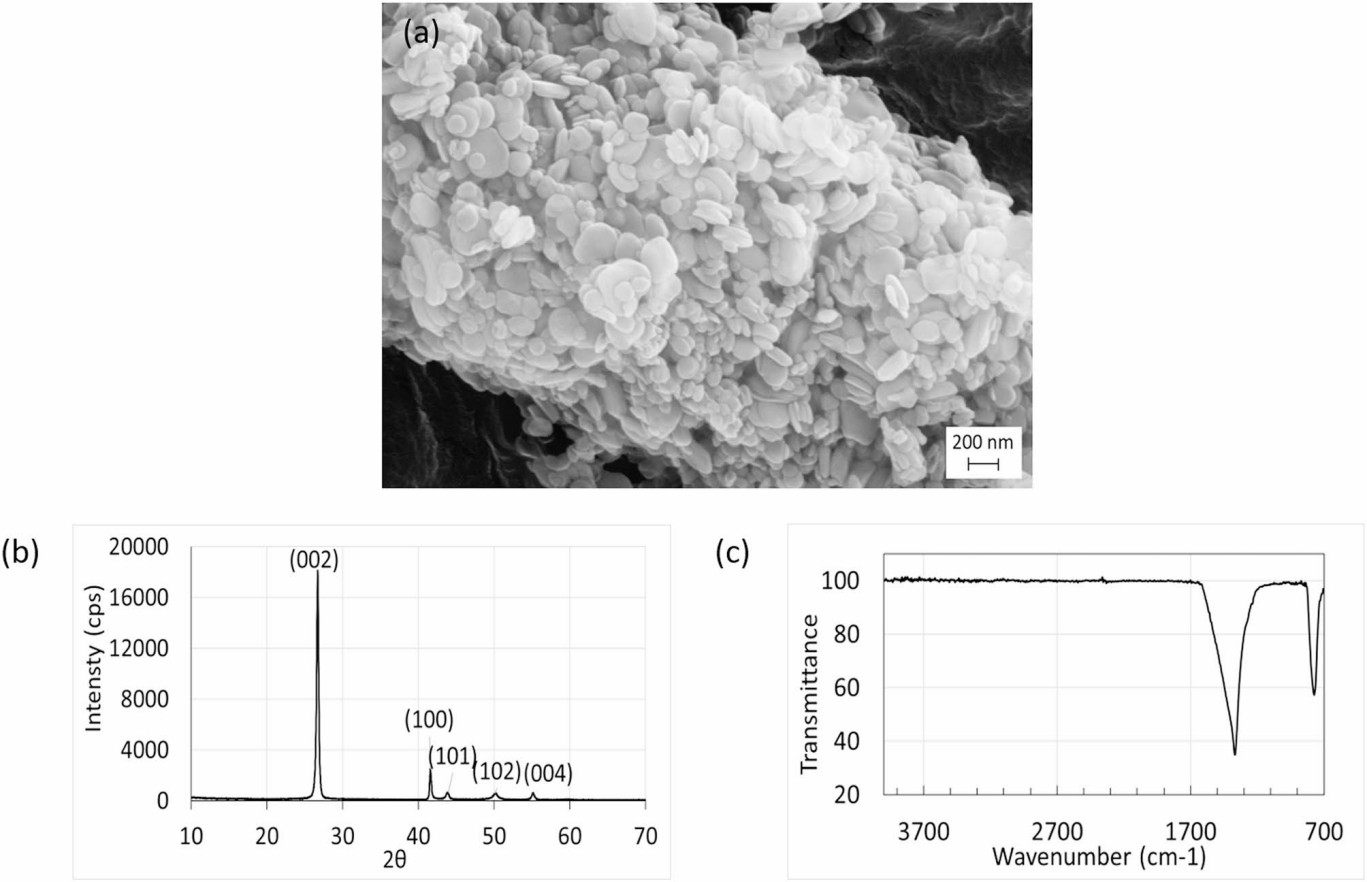
Physicochemical characterization of hBN nanoparticles. **a** SEM image of hBN nanoparticles. **b** XRD pattern of hBN nanoparticles. **c** FTIR spectra of hBN nanoparticles

The SEM images of the hBN particles used in the experimental studies reveal that the particles exhibit a round and platelet-like morphology, with diameters ranging from 50 to 200 nm and thicknesses between 50 and 75 nm (Fig. 1a). The XRD pattern presented in Fig. 1b shows sharp and well-defined diffraction peaks corresponding to the (002), (100), (101), (102), and (004) crystallographic planes, in agreement with the JCPDS card no. 034 − 0421. The presence of these sharp peaks confirms the highly crystalline nature of hBN. In the FTIR analysis, two characteristic absorption bands were observed at 1368 cm⁻¹ and 744.5 cm⁻¹, corresponding to B–N–B stretching vibrations and B–N bending vibrations, respectively (Fig. 1c). The absence of additional peaks in the spectrum indicates the high purity of the hBN particles used.

### HGF cells preserved their cytoskeletal integrity after isolation and during culture

**Fig. 2:**
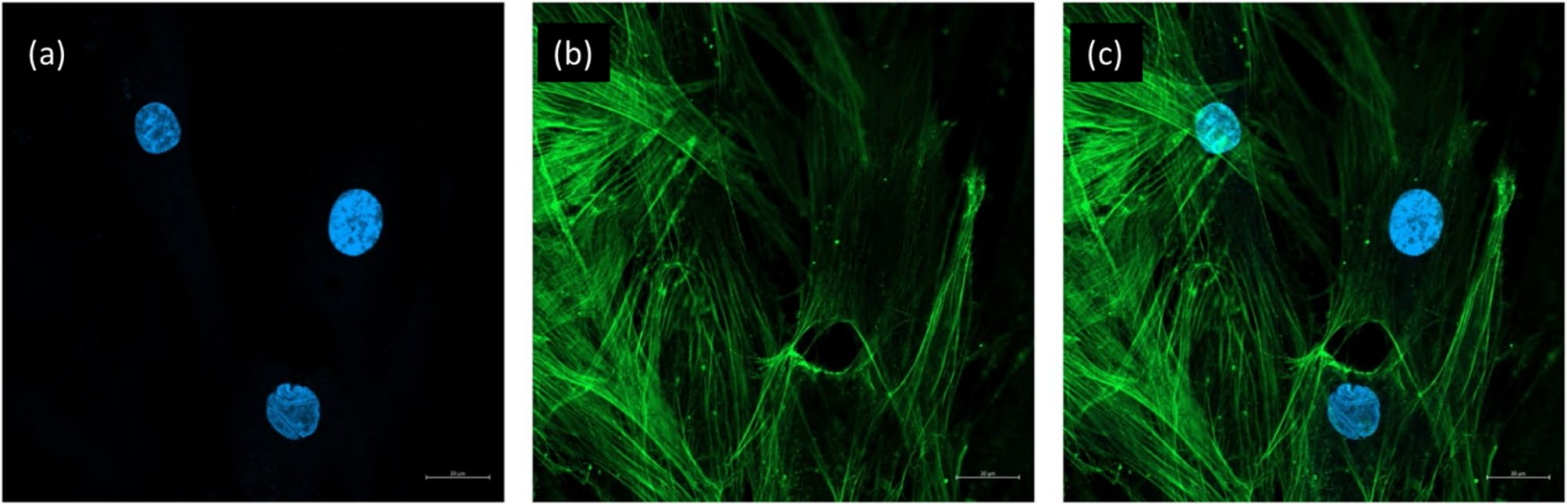
Observation of the cytoskeletal structure of HGF cells by phalloidin staining. **a** DAPI staining. **b** Phalloidin staining. **c** Merge image

The results of F-actin staining clearly showed that HGF cells displayed a well-organized and intact cytoskeletal network. The cells exhibited clear, filamentous structures indicative of proper F-actin organization. The staining revealed a pronounced actin filament pattern throughout the cytoplasm, which extended into the cell periphery, facilitating cell adhesion and spreading (Fig. 2). These findings confirm that the cells maintained their cytoskeletal integrity post-isolation and culture, demonstrating that HGF cells are viable for further experiments.

### The concentration of H_2_O_2_ for the HGF cell injury model was set at 800 µM

**Fig. 3:**
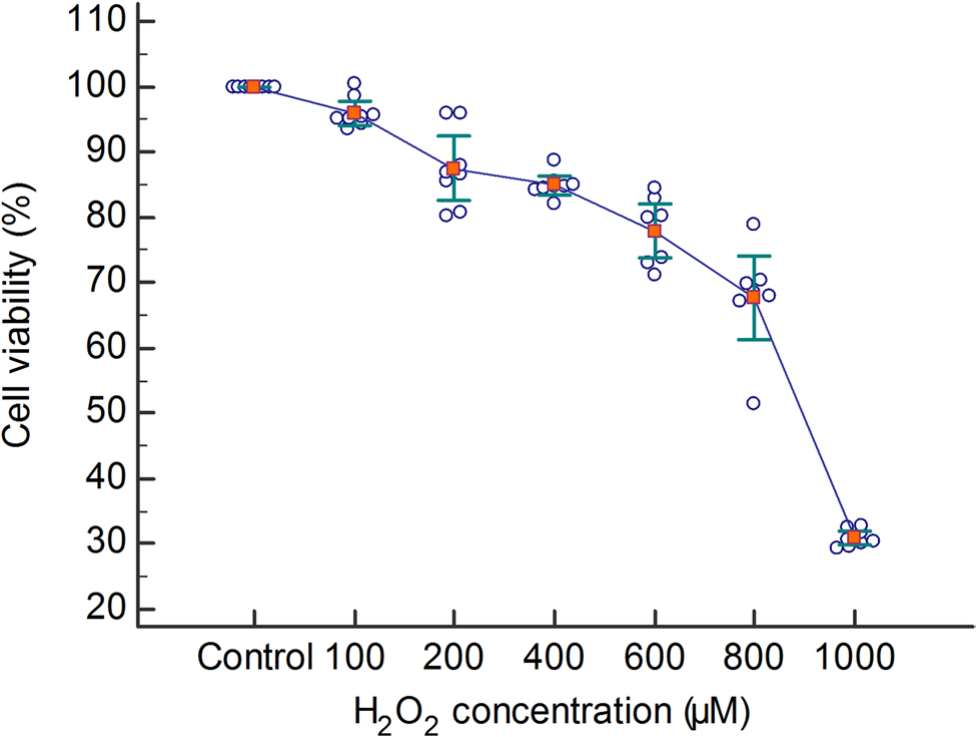
The effect of H_2_O_2_ on cell viability in HGF cells. Data are presented as the mean ± standard deviation (*n* = 8)

The determination of H₂O₂ concentration for the establishment of oxidative damage in HGF cells revealed that exposure to oxidative stress resulted in an increase in cell death for the concentration levels (200, 600, 800, and 1000 µM) (*P* < 0.001) (Table 1; Fig. 3). Based on the XTT assay results, 800 µM was selected for further experiments as it induced a reproducible and moderate level of cytotoxicity (~ 35–40% cell death).

**Table 1:** Cell viability of HGF cells exposed to different concentrations of H₂O₂

Group	Cell viability (%)	*P*_1_	*P*_2_
Control	100 ± 0.00	NA	NA
100 µM	95.96 ± 2.30	0.246	0.485
200 µM	87.48 ± 5.88	**< 0.001**	**0.004**
400 µM	84.88 ± 1.86	**< 0.001**	0.879
600 µM	77.90 ± 4.85	**< 0.001**	**0.028**
800 µM	67.67 ± 7.55	**< 0.001**	**< 0.001**
1000 µM	30.84 ± 1.33	**< 0.001**	**< 0.001**

*P*_1_: post hoc test *P*-values comparing each concentration with the control

*P*_2_: post hoc test *P*-values comparing each concentration with the previous concentration. Data are presented as mean ± standard deviation. *P*-value < 0.05 was considered statistically significant

### hBN concentrations of 0.001 to 0.1 mg/mL did not decrease HGF cell viability

**Fig. 4:**
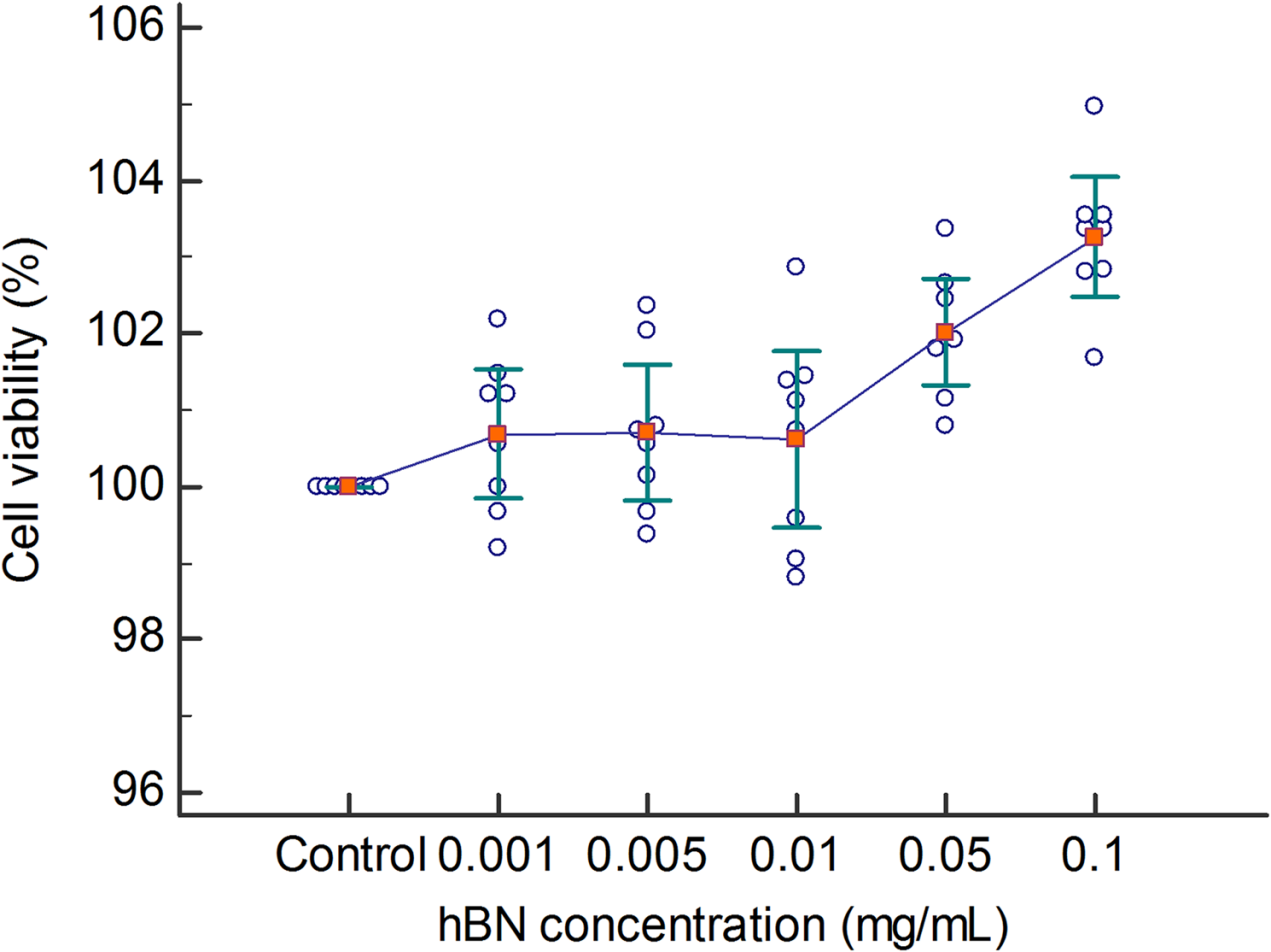
The effect of hBN on cell viability in HGF cells. Data are presented as the mean ± standard deviation (*n* = 8)

When hBN was administered to HGF cells at concentrations of 0.001, 0.005, 0.01, 0.05, and 0.1 mg/mL, none of the concentrations caused cellular damage when compared to the control group. In fact, a relative increase in cell viability was observed at concentrations of both 0.05 mg/mL and 0.1 mg/mL hBN compared to the control (*P* < 0.001). This increase is likely due to the formation of a precipitate at these concentrations, which leads to a color change on the plate that can be measured by spectrophotometry (Table 2; Fig. 4).

**Table 2:** Cell viability of HGF cells exposed to different concentrations of hBN

Group	Cell viability (%)	*P*_1_	*P*_2_
Control	100 ± 0.00	NA	NA
0.001 mg/mL	100.69 ± 1.01	0.479	0.711
0.005 mg/mL	100.71 ± 1.05	0.451	> 0.999
0.01 mg/mL	100.62 ± 1.38	0.575	> 0.999
0.05 mg/mL	100.02 ± 0.82	**0.001**	0.062
0.1 mg/mL	103.26 ± 0.93	**< 0.001**	0.124

*P*_1_: post hoc test *P*-values comparing each concentration with the control

*P*_2_: post hoc test *P*-values comparing each concentration with the previous concentration. Data are presented as mean ± standard deviation. *P*-value < 0.05 was considered statistically significant

### hBN demonstrated protective and therapeutic effects against oxidative damage in HGF cells

**Fig. 5:**
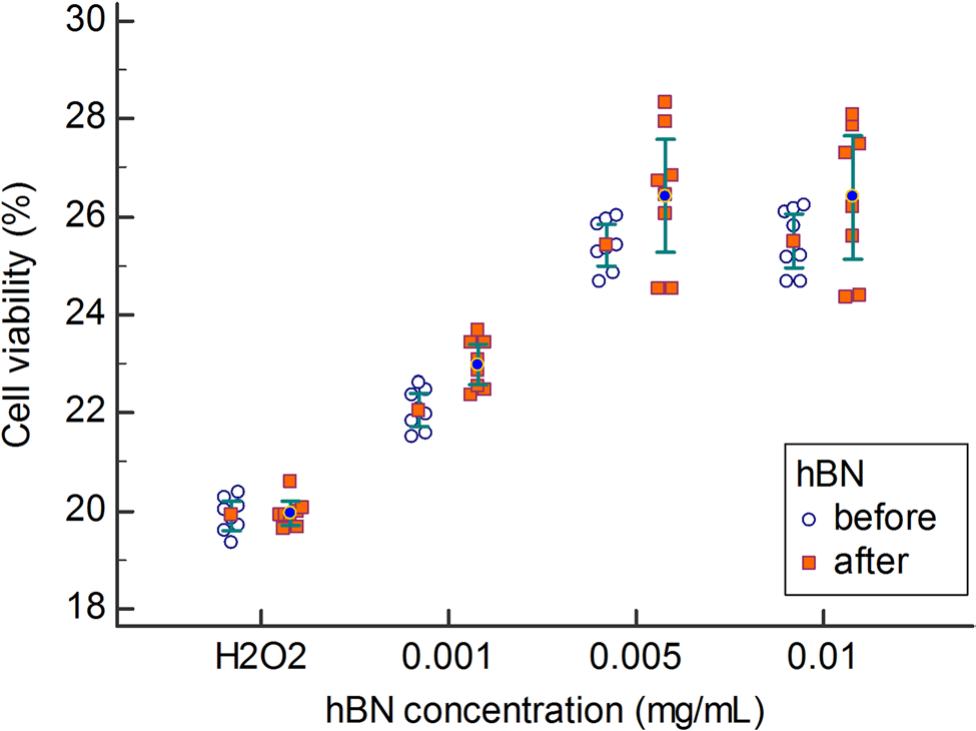
The effect of hBN on cell viability before and after the induction of oxidative damage in HGF cells. Data are presented as the mean ± standard deviation (*n* = 8)

No significant interaction was found between hBN concentration and application time (*P* = 0.334), indicating that the effect of the hBN concentration did not depend on application time. However, there was a significant main effect of hBN concentration *(P* < 0.001), indicating that overall cell viability values differed across concentrations. A significant main effect of application time was also found (*P* = 0.001), suggesting that cell viability values changed across application time regardless of group. Although no significant interaction was observed, exploratory post hoc analyses, with adjustments for multiple testing using Bonferroni correction, were performed within each application time to further examine specific differences between hBN concentrations (Table 3; Fig. 5).

**Table 3:** Cell viability of HGF cells treated with different concentrations of hBN before and after H₂O₂-induced oxidative stress

Cell viability (%)			*P*_1_	*P*_2_	*P*_1_	*P*_2_
Group	Before H_2_O_2_	After H_2_O_2_	Before H_2_O_2_		After H_2_O_2_	
H_2_O_2_ only	19.91 ± 0.35	19.97 ± 0.30	NA	NA	NA	NA
0.001 mg/mL	22.06 ± 0.41	22.99 ± 0.50	**< 0.001**	**< 0.001**	**< 0.001**	**< 0.001**
0.005 mg/mL	25.42 ± 0.50	26.43 ± 1.39	**< 0.001**	**< 0.001**	**< 0.001**	**< 0.001**
0.01 mg/mL	25.50 ± 0.65	26.41 ± 1.51	**< 0.001**	0.999	**< 0.001**	0.999

*P*_1_: post hoc test *P*-values comparing each hBN concentration with the H_2_O_2_ only group

*P*_2_: post hoc test *P*-values comparing each hBN concentration with the previous concentration. Data are presented as mean ± standard deviation. *P*-value < 0.05 was considered statistically significant

When hBN was applied at concentrations of 0.001 to 0.1 mg/mL to HGF cells before the formation of oxidative damage induced by 800 µM H_2_O_2_, a statistically significant increase in cell viability was observed at all concentrations compared to the H_2_O_2_ group (*P* < 0.001). Specifically, 0.001 mg/mL of hBN provided approximately a 2% protective effect against cell damage, while concentrations of 0.005 mg/mL and 0.01 mg/mL offered approximately a 5.5% protective effect against the same oxidative damage (Table 3; Fig. 5).

After the formation of oxidative damage, treatment with hBN at concentrations ranging from 0.001 to 0.1 mg/mL in HGF cells resulted in a statistically significant increase in cell viability compared to the H_2_O_2_ group across all concentrations (*P* < 0.001). Specifically, 0.001 mg/mL of hBN reduced cell damage by approximately 3%, while 0.005 mg/mL and 0.01 mg/mL of hBN led to a reduction in cell damage of approximately 6.5%. (Table 3; Fig. 5).

### According to the TOS results, it was observed that the oxidative mechanism did not play a role in the protective and therapeutic effects induced by hBN

**Fig. 6:**
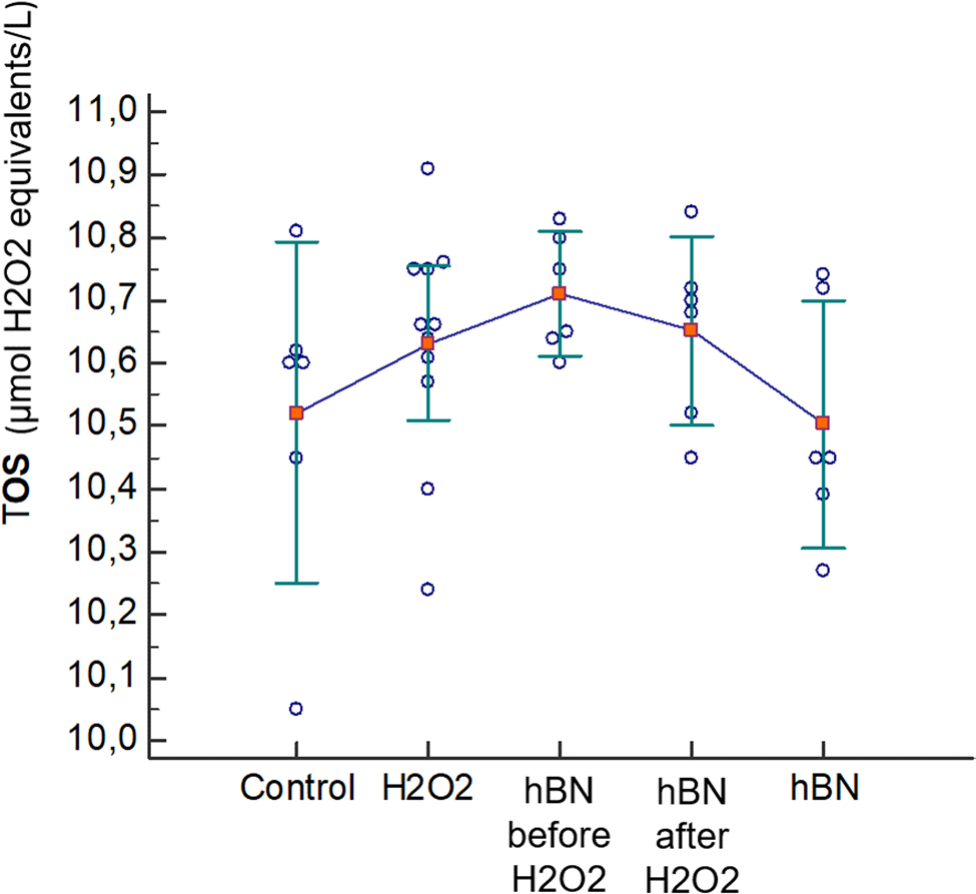
Effect of hBN on TOS levels in HGF cells. Data are presented as the mean ± standard deviation (*n* = 6)

When hBN was used at 0.01 mg/mL either before or after H_2_O_2_-induced oxidative damage in HGF cells, there was no significant change in TOS levels (*P* = 0.234) (Table 4; Fig. 6).

**Table 4:** TOS levels in HGF cells treated with hBN before and after H₂O₂-induced oxidative stress

Group	TOS (µmol H_2_O_2_ equivalents/L)	*P*
Control	10.52 ± 0.26	0.234
H_2_O_2_ only	10.63 ± 0.18	
hBN before H_2_O_2_	10.71 ± 0.09	
hBN after H_2_O_2_	10.65 ± 0.14	
hBN only	10.50 ± 0.19	

*P*: *P*-value of one-way ANOVA. Data are presented as mean ± standard deviation

## Discussion

In this study, we investigated the effects of hBN on cell viability and its protective and therapeutic roles against oxidative damage in HGF cells. Our findings indicated that hBN did not reduce cell viability in HGF cells at any of the concentrations tested (ranging from 0.001 to 0.1 mg/mL). When hBN was administered to HGF cells prior to exposure to oxidative stress, it reduced cell damage by 2-5.5% at concentrations of 0.001–0.01 mg/mL. Furthermore, when hBN was applied to HGF cells after oxidative damage occurred, it also decreased cell damage by approximately 3-6.5% at the same concentrations.

In this study, the selection of hBN concentrations was guided by the findings of Kivanc et al. [31]. Their study on the effects of hBN nanoparticles on human dermal fibroblasts reported no cytotoxicity at concentrations between 0.025 and 0.1 mg/mL. Accordingly, we initially started our experiments with a concentration of 0.1 mg/mL hBN. However, precipitation of hBN was observed at 0.05 and 0.1 mg/mL. Therefore, the concentrations were reduced to 0.001, 0.005, and 0.01 mg/mL for subsequent experiments to ensure solution stability and avoid potential interference with spectrophotometric measurements.

To our knowledge, this study is the first to investigate the effects of hBN on cell viability in HGF cells. However, previous research has examined hBN’s impact on other cell types [31, 32, 40, 41]. In a study that assessed the application of hBN nanoparticles on human dermal fibroblast cell viability, hBN was administered at concentrations ranging from 0.025 mg/mL to 0.4 mg/mL, with cell viability measured at both 24 and 48 h [31]. Notably, human dermal fibroblast cells exposed to low concentrations of hBN (0.025-0.1 mg/mL) exhibited no significant changes in viability. In contrast, at higher concentrations (0.2 mg/mL and 0.4 mg/mL), cell viability decreased by approximately 11–23% on day 1 and 25–37% on day 2. In an another study involving human dermal fibroblasts and human umbilical vein endothelial cells (HUVEC), hBN and BA were administered at concentrations ranging from 10 µg/mL to 500 µg/mL to assess their effects on cell viability [32]. The application of hBN to human dermal fibroblast cells at concentrations up to 100 µg/mL did not result in a significant impact on cell viability; however, higher concentrations (150–500 µg/mL) led to a 64% reduction in cell viability in a dose-dependent manner. Conversely, low doses of hBN (10–100 µg/mL) applied to HUVEC cell lines enhanced cell viability by as much as 118%. While 150 µg/mL and 200 µg/mL of hBN showed no significant effect on cell viability, the highest concentrations (300–500 µg/mL) resulted in a significant decrease in cell viability by 42%. In a study involving osteoblast cell lines, the effects of hBN nanoparticles at concentrations ranging from 20 µg/mL to 200 µg/mL on cell viability were evaluated over three time points: days 1, 3, and 5 [40]. At all concentrations tested, no significant decrease in cell viability was observed, except at the higher concentrations of 50 µg/mL and 200 µg/mL. Both of these higher concentrations resulted in a significant reduction in cell viability. In our study, we observed that hBN did not induce any cell damage when applied at five different concentrations ranging from 0.001 to 0.1 mg/mL in HGF cells. A review of the literature indicates that the doses chosen and the results obtained in our study align well with those reported in research involving various other cell lines.

In this investigation, we assessed the effects of hBN application on HGF cells, both prior to and following the establishment of an inflammation model induced by H_2_O_2_. While we did not find any studies directly comparable in the literature, there are existing researchs that investigate the effects of hBN and various boron compounds in different cell types or inflammation models [25, 32, 33, 40, 42–45]. Sen et al. investigated the effects of hBN and BA on ROS formation in human dermal fibroblast and HUVEC cells at concentrations of 25 µg/mL to 200 µg/mL [32]. They found that treatments at 100 µg/mL and 200 µg/mL significantly reduced ROS levels compared to controls. Cakir et al. assessed the prophylactic effects of hBN nanoparticles on sepsis-induced neurodegeneration [33]. They administered hBN at doses of 50 µg/kg and 100 µg/kg to rats 24 h before inducing inflammation with lipopolysaccharides. The results indicated that hBN treatment significantly decreased TOS and ROS levels, with the 100 µg/kg dose showing the greatest efficacy. Rasel et al. evaluated the effects of hBN on ROS production in osteoblast cell lines at concentrations between 10 µg/mL and 50 µg/mL [40]. Their results showed consistent ROS levels across all concentrations compared to a control group, indicating that hBN nanoparticles do not induce additional oxidative stress and suggest cytocompatibility. On the other hand, Kar et al. investigated the acute cytotoxicity of hBN nanoparticles in animal models, focusing on oxidative stress measurements using the thiol-disulfide bond balance as a biomarker [42]. Following intravenous administration of hBN nanoparticles at concentrations from 50 µg/kg to 3200 µg/kg in rats, blood and tissue analyses indicated that high concentrations of hBN nanoparticles disrupted thiol/disulfide homeostasis, induced oxidative stress through inflammatory responses, and exhibited dose-dependent toxic effects. In our study, we observed that hBN reduced oxidative stress-induced cell damage in HGF cells. However, we found that the application of hBN alone, whether administered before or after oxidative damage, did not lead to a significant change in TOS levels in HGF cells. This indicates that while hBN has protective and therapeutic effects against oxidative damage in HGF cells, it does not exert these effects through TOS. It is believed that this may be due to the fact that the concentrations of hBN showing antioxidant or toxic effects in other studies were significantly higher than those used in our study. When evaluated alongside existing literature, our findings suggest that hBN may exert its protective and therapeutic effects via modulation of ROS-mediated cellular responses rather than direct alteration of total oxidant status. As schematically presented in various publications, the cross-regulation between nuclear factor erythroid 2–related factor 2 (Nrf2) and NF-κB under oxidative stress provides a mechanistic framework supporting this interpretation [46–48]. Nrf2 is a master regulator of antioxidant defenses, activating the expression of cytoprotective enzymes through binding to antioxidant response elements under oxidative challenge [46]. Conversely, NF-κB orchestrates pro-inflammatory and pro-apoptotic gene expression upon activation by ROS or cytokines [46]. Importantly, both pathways have been implicated in periodontal inflammation and tissue destruction. Periodontitis is associated with suppressed Nrf2 activity and enhanced NF-κB signaling, leading to increased oxidative damage and inflammatory mediator production in gingival tissues [6, 47–49]. Thus the capacity of hBN to simultaneously dampen ROS-induced apoptosis and inflammation without significantly altering total oxidant status may reflect its modulatory effects on Nrf2 and NF-κB pathways in gingival fibroblasts. However, further mechanistic studies are warranted to validate these hypotheses in HGF cells.

In addition to their antioxidant effects, hBN nanoparticles have demonstrated promising antibacterial and anti-inflammatory properties, which are critical in the pathogenesis of periodontal diseases. Recent studies have shown that hBN inhibits the growth and biofilm formation of oral pathogens such as *Streptococcus mutans* and *Candida* spp., while maintaining biocompatibility at therapeutic concentrations [31]. Moreover, hBN has been reported to reduce levels of pro-inflammatory cytokines such as TNF-α and IL-1β in experimental inflammation models [33]. Supporting this, Turkez et al. observed enhanced wound healing and antibacterial activity against *Staphylococcus aureus* and *Escherichia coli* in human dermal fibroblast cultures following treatment with alpha-lipoic acid–conjugated hBN nanoparticles [50]. Given that both bacterial colonization and sustained inflammation are hallmark features of periodontal disease, the emerging evidence of hBN’s multifunctional bioactivity underscores its potential as a novel adjunctive agent in periodontal therapy.

## Limitations

To the best of the authors’ knowledge, this study constitutes the first in vitro investigation into the protective and therapeutic effects of hBN against oxidative damage in HGF cells. The findings provide preliminary support for the potential application of hBN in mitigating oxidative stress–mediated inflammation and tissue injury relevant to periodontal diseases. Nevertheless, several limitations should be acknowledged when interpreting these results. First, the use of a single-donor primary HGF cell line may not fully capture interindividual biological variability, which could influence cellular responses to oxidative insults and nanomaterial exposure. Although primary cells offer superior physiological relevance compared to immortalized cell lines, the inclusion of cells from multiple donors in future studies, particularly from patients with periodontal disease accompanied by chronic comorbidities such as diabetes and hypertension, would improve the generalizability, biological robustness and clinical relevance of the findings. Furthermore, although it is hypothesized that hBN may exert its effects through modulation of cellular stress response pathways, such as those involving Nrf2 and NF-κB, the current study did not include molecular-level investigations. As a result, the precise biological mechanisms underlying the observed effects remain to be elucidated, highlighting the need for further mechanistic studies. In addition, although the hBN concentration range used was informed by previous cytocompatibility data, it may not comprehensively represent the full therapeutic window of the material. Importantly, even a single and short-term exposure to hBN was sufficient to induce a statistically significant increase in cell viability (approximately 2–6.5%) under oxidative conditions. These findings suggest that repeated or prolonged application might result in more pronounced biological effects. Moreover, the observed increase in cellular viability under stress conditions may have important clinical implications, particularly for patient populations with compromised healing capacity, such as elderly individuals or those with systemic conditions like diabetes. Nonetheless, it is important to acknowledge that the clinical relevance of these in vitro findings remains uncertain. Therefore, future studies should focus on elucidating the dose-dependent biological effects of hBN, verifying its mechanism of action through molecular investigations, and evaluating its efficacy and safety in more complex in vitro and in vivo models.

## Conclusion

This in vitro study provides preliminary evidence regarding the effects of hBN on HGF cells. The findings indicate that hBN does not exert cytotoxic effects at concentrations ranging from 0.001 to 0.1 mg/mL. Moreover, when applied at 0.001, 0.005, or 0.01 mg/mL, either before or after oxidative injury, hBN appears to enhance cell viability under oxidative conditions. However, these results should be interpreted in the context of the study’s design and limitations, including the absence of mechanistic analyses and the inherent limitations of the in vitro model. Therefore, while these findings underscore the potential of hBN in the prevention and management of periodontal diseases, further preclinical and clinical studies are necessary to confirm and extend these findings.

## Data Availability

The datasets used and/or analyzed during the current study are available from the corresponding author on reasonable request.

## Abbreviations

hBN: Hexagonal boron nitride
H_2_O_2_: Hydrogen peroxide
HGF: Human gingival fibroblast
XTT: 2,3-bis-[2-methoxy-4-nitro-5-sulfophenyl]-2 H-tetrazolium-5-carboxyanilide salt
TOS: Total oxidant status
ROS: Reactive oxygen species
NF: Nuclear factor
RANKL: Receptor activator of nuclear factor-κB ligand
BA: Boric acid
BN: Boron nitride
MDCK: Madin-Darby Canine Kidney
TNF: Tumor necrosis factor
IL: Interleukin
SEM: Scanning electron microscope
XRD: X-ray diffraction
ATR-FTIR: Attenuated total reflectance-fourier transform infrared spectroscopy
DMEM: Dulbecco’s modified eagle medium
FBS: Fetal bovine serum
PBS: Phosphate buffered saline
DAPI: 4′,6-diamidino-2-phenylindole
HUVEC: Human umbilical vein endothelial cells
Nrf2: Nuclear factor erythroid 2–related factor 2
